# Deprescribing Anticholinergic Medications in Hospitalised Older Adults: A Systematic Review

**DOI:** 10.1111/bcpt.70103

**Published:** 2025-08-31

**Authors:** Rhianna Griffiths, Steve Lim, Julian Lin, Andrew Bates, Liam Jones, Kinda Ibrahim

**Affiliations:** ^1^ Faculty of Medicine University of Southampton Southampton UK; ^2^ NIHR Applied Research Collaboration Wessex University of Southampton Southampton UK; ^3^ University Hospital Southampton NHS Foundation Trust Southampton UK; ^4^ NIHR Southampton Biomedical Research Centre University Hospital Southampton Southampton UK

**Keywords:** anticholinergic burden, deprescribing, hospital care, medication review, older adults

## Abstract

**Background:**

Anticholinergic medication use is increasing, particularly among older adults due to polypharmacy and comorbidities. High anticholinergic burden is linked to adverse outcomes such as reduced mobility and increased dementia risk. Acute hospital stays may offer an opportunity to address this often‐overlooked issue.

**Aims:**

To examine the effects of deprescribing anticholinergic medications on outcomes in older hospitalised patients.

**Methods:**

Medline, Web of Science, Cochrane Library and Embase were searched from inception to September 2024. Studies included hospital‐based deprescribing or medication review interventions targeting anticholinergic burden in patients aged ≥ 65 years. Narrative synthesis followed SWiM guidelines, with quality assessment using JBI Checklists.

**Results:**

From 2042 records, eight studies met inclusion criteria. Designs included cohort (*n* = 4) and pre‐post quasi‐experimental (*n* = 4), with follow‐up durations of up to 3 months. All reported medication‐related outcomes; four assessed acceptability, one included clinical outcomes, and none examined safety. Six studies reported reductions in anticholinergic burden scores; three showed significant decreases in the proportion of patients prescribed anticholinergics, and two noted fewer potentially inappropriate medications. Most recommended changes were implemented.

**Conclusion:**

Deprescribing interventions in hospital appear acceptable and effective in reducing anticholinergic burden. However, evidence on clinical outcomes, costs and safety is limited. Further RCTs with longer follow‐up are needed.

## Summary

Anticholinergic medicines, often used by older adults with multiple health issues, can increase risks such as memory problems and reduced mobility. Hospital stays may provide a chance to review and reduce these medicines. This review looked at studies where doctors or pharmacists reduced or stopped such drugs for patients aged 65 and over during hospital care. Out of more than 2000 studies, eight were included with follow‐up lasting up to 3 months. Studies found fewer patients were prescribed anticholinergics and other potentially inappropriate medicines. Most of the recommended changes were carried out. While results are promising, more research is needed to understand long‐term effects, costs and safety.

## 1 Introduction

Around 33%–47% of older adults are prescribed one or more medications with anticholinergic effects for conditions such as depression, pain control, psychosis, urinary incontinence and allergic rhinitis [1, 2]. A UK study reported up to a ninefold increase in the anticholinergic burden over 25 years with increases in prescribing of most anticholinergic drug classes and in polypharmacy [3]. Anticholinergic burden—the accumulation of higher levels of exposure due to one or more anticholinergic medications and the attendant increased risk of medication‐related adverse effects [4]—is associated with increased risk of falls, cognitive impairment and dementia, and all‐cause mortality (OR = 1.4, age > 65 years) [5, 6, 7, 8]. Therefore, anticholinergic burden scores are suggested as a potential marker for cognitive decline or as a causative risk factor [9].

Several anticholinergic burden quantification scales have been developed, providing a list of anticholinergic medications and a rank of low to high risk based on anticholinergic activity such as Anticholinergic Cognitive Burden (ACB) scale and anticholinergic effect on cognition scale AEC. There is no universally accepted quantification method to estimate anticholinergic burden at the individual patient level [10]. Existing tools derived from expert consensus limit the quantification of anticholinergic burden as they do not take into consideration the dose and the central nervous system distribution of drugs [11]. Considerable variation exists among anticholinergic risk scales, in terms of selection of specific drugs, as well as of grading of anticholinergic potency. NICE guideline NG97 mentions the ACB scale as a good measure to quantify anticholinergic burden; however, it states that there is insufficient evidence to recommend one over the others [12].

It has been suggested that a medication review and assessment of anticholinergic burden score should be completed for older people on admission to hospital if the admission is related to falls or delirium [13]. The NICE Guideline for Dementia advises addressing reversible causes of cognitive decline, including delirium, depression, sensory impairment [such as sight or hearing loss] or cognitive impairment from medicines associated with increased anticholinergic burden before referral to a specialist dementia diagnostic service [12]. The guideline also advises clinicians to consider minimising the use of medicines associated with increased anticholinergic burden, and if possible, look for alternatives. The hospital setting is an ideal opportunity to identify and target anticholinergic prescribing due to access to multidisciplinary teams and the controlled environment of the inpatient setting facilitating close monitoring of patients' responses. However, few studies have investigated patterns of anticholinergic medications use or deprescribing these medications in a hospital setting. An observational study across different hospitals in the United Kingdom, Finland, the Netherlands, Italy and New Zealand reported no change in anticholinergic scores in hospitalised older people admitted with a diagnosis of delirium, chronic cognitive impairment or falls during their acute admission [14].

Deprescribing has been shown to produce positive health outcomes for older people and result in improved medication adherence and reduced costs [15, 16]. An Australian study projected that if the average number of medications taken per person could be reduced by one, this would result in an annual cost‐saving of $463 million [17]. A recent review of interventions to reduce anticholinergic prescribing errors identified 23 studies, mainly in residential homes and community settings [18]. Barriers and facilitators for deprescribing anticholinergic medications are not well understood, and a recent review found only two papers which reported a lack of collaborative approaches to deprescribing, low confidence among healthcare professionals, lack of system resources and organisation of care as potential barriers [19]. Whereas facilitators included the use of multidisciplinary teams, geriatric case conferences, medication review by pharmacists and the use of information technology to support medication decisions. The aim of this review was to examine the effect of deprescribing interventions targeting anticholinergic burden in hospitalised adults aged ≥ 65 years on medication‐related, clinical and safety outcomes.

## 2 Methods

### 2.1 Data Sources and Searches

Four online databases (Embase, Cochrane Library, Medline and Web of Science) were searched for relevant literature from database inception to September 2024. The search strategy (Appendix A) was created with the help of a senior librarian. The review was registered on the international prospective register of systematic reviews (PROSPERO) ID number: CRD42024592049.

### 2.2 Study Inclusion

The PICOS framework was used to develop the inclusion and exclusion criteria. The study inclusion and exclusion criteria are presented in Table 1.

**TABLE 1:** Study inclusion and exclusion criteria.

PICOS	Inclusion criteria	Exclusion criteria
Population	Older hospitalised patients. Aged 65 years old or older.	People below the age of 65 years old.
Intervention	Any intervention that aims to reduce anticholinergic burden in hospitalised patients through deprescribing or medication review.	Any intervention that involves other intervention components alongside deprescribing/medication review.
Comparator	Any or no comparator.	NA
Outcomes	The primary outcome is the measure of the effects of deprescribing on medication‐related outcomes, clinical outcomes and safety outcomes. Secondary outcomes will include costs and acceptability/feasibility of deprescribing anticholinergic medications.	NA
Study design	Any study design including RCTs and non‐RCTs (e.g., cohort studies, cross‐sectional, pre‐post comparison).	Qualitative studies, systematic reviews.

#### 2.2.1 Types of Studies

Any study design including randomised controlled trials (RCTs) and non‐randomised controlled trials (non‐RCTs), e.g., cohort studies, pre‐post comparison was included. Qualitative studies and systematic reviews were excluded.

#### 2.2.2 Types of Participants

This review was evaluating interventions targeting older hospitalised adults who were aged 65 years or older.

#### 2.2.3 Types of Intervention

This review included any intervention that aimed to reduce anticholinergic burden in patients through deprescribing or medication review that included reducing, stopping or switching drugs. Any interventions that involved other components alongside deprescribing or medication review were excluded if the results could not be attributed to the deprescribing element alone. Interventions had to be within a hospital setting.

#### 2.2.4 Types of Comparators

This review included any study that had any or no comparator.

#### 2.2.5 Types of Outcomes

The primary outcome in this review was the measure of the effects of deprescribing on medication‐related outcomes (including number of medications, anticholinergic burden scores, Drug Burden Index [DBI]), clinical outcomes (including quality of life, cognition, physical function) and safety outcomes (re‐hospitalisation, death, institutionalisation). Secondary outcomes included costs and acceptability/feasibility of deprescribing anticholinergic medications.

### 2.3 Study Selection

Endnote was used by two authors (KI and RG) to independently review articles by first screening titles and abstracts against the inclusion/exclusion criteria, followed by full text review of potentially eligible papers. Any disagreements between the two authors were resolved through discussion. The references of included studies were then searched for further appropriate studies.

### 2.4 Quality Assessment

Joanna Briggs Institute checklist for each study type was used to assess the quality of included studies by three authors independently (RG, SL and JL), and any disagreements were resolved through discussions.

### 2.5 Data Extraction

The relevant data from included studies was extracted into a pre‐designed template on Excel and included: author, year of publication, country, setting, study design, study aim, sample size, characteristics of participants, description of intervention, description of comparator, length of follow up, anticholinergic burden measure, deprescribing tool and outcomes. Data extraction was executed by three authors independently for each study (RG, AB and LJ).

### 2.6 Data Synthesis

Synthesis without meta‐analysis (SWiM) was used for narrative synthesis of the findings. Meta‐analysis was not possible due to the heterogeneity in study design and outcome measures. Studies were grouped according to outcome data [20].

## 3 Results

**FIGURE 1:**
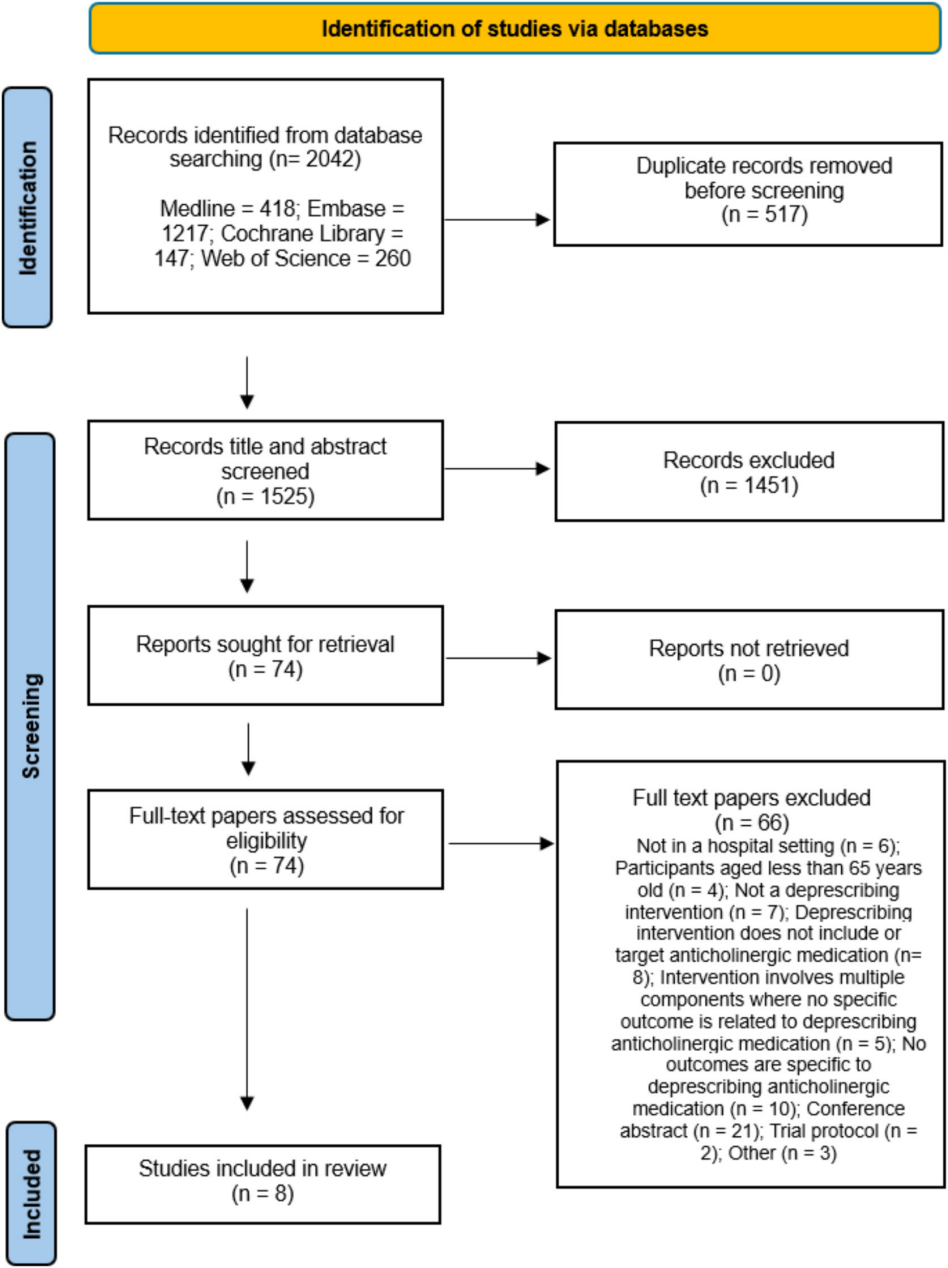
PRISMA flow diagram.

Online searches of the databases retrieved 2042 citations, and eight studies were included in this review (see Figure 1). The inter‐rater agreement on paper screening was 85%; disagreements were resolved through discussion (KI and RG). This review includes eight studies of varying study designs: four cohort studies [21, 22, 23, 24] and four pre‐post quasi‐experimental studies [25, 26, 27, 28]. The studies were performed in six different countries: Australia (*n* = 2) [25, 27], Spain (*n* = 2) [21, 23], Germany (*n* = 1) [22], Italy (*n* = 1) [26], Scotland (*n* = 1) [28] and South Korea (*n* = 1) [24]. The studies were all published between 2013 and 2024 (for more details, see Table 2). The length of follow‐up varied between studies; five of the studies [23, 25, 26, 27, 28] followed patients only until discharge, and the follow‐up in the remaining studies [21, 22, 24] ranged from 2 weeks to 4 months. Study quality varied and was mostly of poor or moderate quality.

**TABLE 2:** Summary of included studies.

First author	Country	Study design	Participants	Intervention	Type of medication review	Comparator	Deprescribing tool/ACB measure	Length of follow‐up	Key outcomes	Quality score
T. Wehran [22]	Germany	Cohort study	*N* = 20	Pharmacists use an algorithm to perform medication review and develop medication change recommendation letters, which are received by physician	Pharmacist‐led	Patients with no anticholinergic load (control group)	Anticholinergic outcome assessment battery (anticholinergic load)	2 weeks	Seven participants in the intervention group (63.6%) had a reduced anticholinergic load. Patients with a reduced anticholinergic load demonstrated a significant improvement in Neuropsychological Assessment Battery memory test scores from baseline to follow‐up compared with those with unchanged medication (6 ± 3 vs. −1 ± 6 points).	8/9
K. Fujita [25]	Australia	Pre‐post quasi‐experimental study	*N* = 409, median age was 89.0 (IQR 86–92) years	The use of a DBI intervention bundle and a stewardship phase	Pharmacist‐led	Control group (standard care) and stewardship group	Drug Burden Index	At discharge	The proportion of patients who had at least one DBI‐contributing medication stopped or dose reduced on discharge increased from 29.9% (43/144) in the control period to 37.5% (66/176) in the intervention period and 43.1% (59/137) in the stewardship period. Using the control period as the reference, the ‘stewardship programme’ significantly increased the proportion of medications stopped or reduced compared with the control period (aRD 12.1%, 95% CI 1.0%–24.0%), while the ‘intervention bundle’ alone showed no significant effect (aRD 6.5%, 95% CI −3.2% to 17.5%).	8/11
M. Espaulella‐Ferrer [21]	Spain	Prospective cohort study	*N* = 150, mean age 89 ± 4.5 years	Medication review applied to the patient‐centred prescription (PCP) model	MDT‐led	No comparator	Drug Burden Index	3 months	After 3 months, the mean DBI score decreased significantly from 1.06 ± 0.7 to 0.95 ± 0.7 (*p* < 0.001).	8/9
H.S. Tay [28]	Scotland	Pre‐post quasi‐experimental study	*N* = 140	List of drugs with ARS score put on ward trolley and consultant made aware of the ARS score of patients before medication review	Consultant‐led	Standard care	Anticholinergic drug exposure (number of anticholinergic drugs and Anticholinergic Risk Scale [ARS] score)	At discharge	Consultant medication review reduced ARS scores (*p* = 0.01), especially following the introduction of the information system (*p* = 0.002).	7/11
S. Ghibelli [26]	Italy	Pre‐post quasi‐experimental study	*N* = 134, mean age 81.3 years.	Use of a computer‐based application (INTERcheck) to review pharmacological profiles	Computerised prescription support system	Standard care	ACB scale	At discharge	In the observation phase, 29 (39.1%) patients were exposed to at least one PIM on admission and 28 (37.8%) at discharge; the mean number of PIMs per patient was similar on admission (0.5) and at discharge (0.4). In the intervention phase, 25 (41.7%) patients were exposed to at least one PIM at admission, and 7 (11.6%) at discharge (*p* < 0.001). Similarly, the mean number of PIMs per patient significantly decreased at discharge from 0.5 to 0.1 (*p* < 0.001)	8/11
N. Masnoon [27]	Australia	Pre‐post quasi‐experimental study	*N* = 256, median patient age = 87 (IQR 82–91) years	The use of a DBI intervention bundle and a steward to promote and increase clinician engagement	Pharmacist‐led	No comparator	Drug Burden Index	At discharge	The steward made 170 recommendations for 117 patients. Registrars agreed with 141 recommendations (82.9%) for 95 patients (81.2%) and actioned 115 deprescribing recommendations for 80 patients.	6/11
M. Hernandez [23]	Spain	Cohort study	*N* = 65, mean age 84.9 ± 6.7 years	Medication review by pharmacist, recommendations sent to physician via email, telephone or a weekly meeting, followed by MDT follow‐up	Pharmacist‐led	No comparator	Drug Burden Index	At discharge	86.9% of the proposed interventions or recommendations were accepted by the physician. The mean (SD) anticholinergic burden per patient reduced from 1.38 (0.7) pre‐intervention to 1.08 (0.7) post intervention (*p* < 0.016). The number of patients who presented with an anticholinergic burden > 1 reduced from 44 (DBI range 0.3–3) to 30 (DBI range 0.3–2.6).	7/9
A. J. Kim [24]	South Korea	Cohort study	*N* = 95, mean age of the patients was 74.9 ± 7.3 years	Pharmacist‐led geriatric medication management service to improve the quality of medication use, followed by recommendation letters	Pharmacist‐led	No comparator	Korean Anticholinergic Burden Scale and Beers Criteria 2019	4 months after study completion	Following the intervention, the total number of medications and potentially inappropriate medications (PIMs) decreased from 13.5 ± 4.3 to 10.9 ± 3.8 and 1.6 ± 1.4 to 1.0 ± 1.2, respectively (both *p* < 0.001). The proportions of patients on any and two or more strong anticholinergic drugs reduced from 34.5% to 20.7% and 4.6% to 2.3%, respectively (*p* = 0.003). The anticholinergic burden score, determined using KABS, decreased from 2.7 ± 2.6 at baseline to 1.8 ± 2.2 (*p* < 0.001).	5/9

Abbreviations: aRD = adjusted risk difference, ARS = Anticholinergic Risk Scale, DBI = Drug Burden Index, MDT = multi‐disciplinary, PIMs = potentially inappropriate medications.

Sample sizes ranged from 20 to 409 patients. The percentage of women in each study varied from 41% to 93%. The mean age of participants ranged between 75 and 89 years; however, the mean age of participants was only reported in six out of the eight studies [21, 22, 23, 24, 26, 28]. One study looked at deprescribing only in patients with dementia [23] and another study looked at deprescribing only in patients with reduced kidney function [24].

All eight studies included a medication review as the primary part of the intervention. The majority (*n* = 5) of the studies had a pharmacist‐led medication review [22, 23, 24, 25, 27]. The other studies included a consultant‐led medication review (*n* = 1) [28], multi‐disciplinary team (MDT) led medication review (*n* = 1) [21] and a computerised prescription support system medication review (*n* = 1) [26]. Three studies [24, 25, 27] involved other elements alongside medication review in the intervention: two studies [25, 27] involved an education module for staff on polypharmacy in older patients, two studies [25, 27] involved leaflets on deprescribing that were given to patients and carers, and one study [24] involved patient counselling. Patient counselling was performed by a pharmacist; the pharmacist would talk to a patient about their medication history, deprescribing and precautions that are needed when on medications [24]. Five different methods of measuring anticholinergic burden were used across the studies. Four studies [21, 23, 25, 27] used the DBI; one study [22] used anticholinergic load (calculated by the number of drugs with strong anticholinergic activity according to a list developed by the study team); one study [28] used anticholinergic drug exposure (calculated using the number of anticholinergic drugs and ARS); one study [26] used the ACB scale; and one study [24] used both the Korean Anticholinergic Burden Scale (KABS) and Beers Criteria 2019.

### 3.1 Studies Outcomes

#### 3.1.1 Medication‐Related Outcomes

##### 3.1.1.1 Anticholinergic Medication Outcomes

Seven of the eight studies reported an outcome related to changes in anticholinergic medications. The outcomes varied across the studies. Six studies reported a reduction in anticholinergic burden score post intervention using different scales/scores such as DBI (*n* = 2), ACB scale (*n* = 1), anticholinergic load (*n* = 1), ARS (*n* = 1) and KABS (*n* = 1) [21, 22, 23, 24, 26, 28] For example, Kim et al. showed that the anticholinergic burden score, determined using KABS, decreased from 2.7 ± 2.6 at baseline to 1.8 ± 2.2 (*p* < 0.001) [24]. Four studies found a significant increase in the proportion of patients who had anticholinergic medications reduced or stopped following the intervention [22, 24, 28]. A study by Werhan et al. reported that 63.6% of patients in the intervention group had reduced anticholinergic load following the intervention [22]. Tay et al. reported that the proportion of patients on anticholinergic medications that were either stopped or reduced increased from 35% in the control phase to 72% in the intervention phase [28]. Fujita et al. reported that the proportion of patients with anticholinergic medications reduced or stopped increased from 29.9% in the control period to 43.1% in the stewardship phase (intervention phase alone, was not significant) [25]. Kim et al. reported that the proportions of patients on any and two or more strong anticholinergic drugs reduced from 34.5% to 20.7% and 4.6% to 2.3%, respectively (*p* = 0.003) between baseline and following the pharmacist‐led intervention [24].

Two studies reported the number of anticholinergic medications taken by patients [25, 28]. Tay et al. showed a greater reduction in the total number of anticholinergic medications being prescribed in the intervention phase compared with standard care. The number of anticholinergic medications prescribed reduced from 29 to 20 in standard care but reduced from 24 to 11 in the intervention phase [28]. Fujita et al. found that in certain classes of anticholinergic medications (opioids, antiepileptics and antipsychotics) the proportion of DBI‐contributing medications deprescribed was greater in the intervention and stewardship phase compared with the control phase. During the control period, usual care was provided. During the intervention, access to the intervention bundle was added, including a clinician interface displaying DBI score in the electronic medical record. In a subsequent ‘stewardship’ period, a stewardship pharmacist used the bundle to provide clinicians with patient‐specific recommendations on deprescribing DBI‐contributing medications. The percentage of opioids deprescribed in the control phase was 18%, in the intervention phase was 35%, and in the stewardship phase was 46% [25]. The percentage of antiepileptics deprescribed in the control phase was 7%, in the intervention phase was 22%, and in the stewardship phase was 25% [25]. The percentage of antipsychotics deprescribed in the control phase was 53%, in the intervention phase was 54%, and in the stewardship phase was 60% [25].

Of the seven studies, only three studies had a post‐intervention follow‐up ranging from 2 weeks to 4 months [21, 22, 24]. All reported that the reduction in anticholinergic burden score had been maintained during the follow‐up period.

##### 3.1.1.2 Potentially Inappropriate Medications (PIMs)

Two studies reported on the change in prevalence of PIMs and mean number of PIMs before and after an intervention [24, 26]. PIMs were measured in different ways across the studies. Ghibelli et al. used the INTERcheck software, which contains explicit criteria on PIMs based on Beer's Criteria 2003. Whereas, Kim et al. used Beer's Criteria 2019. Both studies found that there was a significant reduction of 0.5 (*p* < 0.001) in the mean number of PIMs per patient following the intervention [24, 26]. Ghibelli et al. reported that in the intervention phase, on admission, 41.7% of patients were exposed to one or more PIMs compared with 11.6% at discharge (*p* < 0.001).

#### 3.1.2 Clinical‐Related Outcomes

Only one study (Wehran et al.) which involved a medication review and recommendation letters reported on clinical‐related outcomes and found that there was a significant improvement in the memory score (using the Neuropsychological Assessment Battery) of patients who had reduced their anticholinergic load during the intervention compared with the patients who had not or patients in the control group (*p* < 0.05). Improvements were also seen in the attention score of patients who reduced their anticholinergic load; however, the results were not significant [22].

#### 3.1.3 Acceptability‐Related Outcomes

Four out of the eight studies reported on the acceptance rate of the medication change recommendations by clinicians following a medication review [21, 23, 24, 27]. Acceptance rates ranged from 79.4% to 86.9% across the studies [21, 23, 24, 27]. Recommendations made by pharmacists were communicated to the medical team or physician within the hospital [23, 24, 27] or general practitioner via a discharge summary and followed up the recommendations 3 months later [21]. The study which yielded the highest acceptance rate communicated the recommendations to a physician within the hospital via email or phone call, followed by a weekly meeting to discuss the recommendations (86.9%) [23].

Three of the eight studies discussed the reasons why medication recommendations for deprescribing anticholinergic medications were not implemented [22, 27, 28]. The reasons fell into three broad categories: patient‐related factors, clinical considerations and system‐level challenges. Patients fear of symptom recurrence, previous failed attempts, concerns about withdrawal or a desire to avoid disrupting discharge plans were reported. Clinically, deprescribing was perceived as inappropriate if the patient was acutely unwell or had ongoing psychiatric needs. System‐level barriers included difficulties accessing the general practitioner or unclear prescribing rationale, such as an incorrect or outdated indication.

## 4 Discussion

This is the first systematic review exploring deprescribing interventions to reduce anticholinergic burden specific to older hospitalised adults, highlighting limited research in this area. Previous reviews have evaluated interventions to reduce anticholinergic burden in older adults but not exclusively in a hospital setting [29]. All interventions involved a medication review, mainly led by pharmacists, with some including other components such as staff teaching, patient leaflets, patient counselling and a stewardship period. The most reported outcomes in the included studies were medication‐related (e.g., anticholinergic burden score and number of PIMs) which were shown to significantly improve following the intervention. The studies reported high acceptance rates of pharmacists' recommendations (> 80%), suggesting that the interventions were acceptable. No studies reported on safety or cost outcomes, and only one study reported the impact of the intervention on clinical outcomes (i.e., cognition).

The majority of data extracted from the included studies related to medication outcomes, though there was considerable variation in how these were measured. Four different anticholinergic burden scales were used: the DBI, ACB scale, Anticholinergic Risk Scale (ARS) and KABS. Prior research has highlighted inconsistencies between these tools, noting variation both in the quantification of anticholinergic burden and in the drugs classified as anticholinergic agents [30, 31]. Despite the use of different scales, all studies reported a reduction in anticholinergic burden following intervention. However, the broader literature presents mixed findings [29, 32]. For instance, Braithwaite et al.'s review found no significant difference in ACB scores between intervention and control groups in community‐based studies [33]. In contrast, Nakham et al.'s review included eight papers and reported positive effects across multiple settings, particularly in hospitals, where all four included studies showed significant reductions in ACB score among older adults [33]. This suggests that setting may influence the success of interventions aimed at reducing anticholinergic burden, with hospital‐based approaches potentially being more effective.

The most notable finding of this review was the lack of data on safety and clinical outcomes in the included studies. Considering the evidence of the cognitive and physical impacts of anticholinergic burden on function and cognition, it is surprising that only one study included in the review considered clinical outcomes. One review assessed the efficacy and safety of anticholinergic medication reduction interventions for improving cognitive outcomes in cognitively healthy older adults and older adults with pre‐existing cognitive issues in nursing homes and the community. The review identified three randomised trials and reported insufficient evidence to reach any conclusions on the effects of anticholinergic burden reduction interventions on cognitive outcomes and other clinical outcomes such as mortality, quality of life, clinical global impression, physical function, institutionalisation, falls, cardiovascular diseases or neurobehavioural outcomes [34]. This highlights the current limited research in deprescribing anticholinergic medications and the need for further large‐scale well‐designed trials with longer follow‐up periods to test the safety and effects on clinical outcomes.

## 5 Strengths and Limitations

A key strength of this review is that this is the first review performed to systematically evaluate the effects of deprescribing interventions on anticholinergic burden in older hospitalised adults. The review has identified key gaps in research including poor reporting of clinical outcomes (e.g., cognition, falls and functional status) and short durations of follow‐up. However, there are a few limitations to this review; for instance, only papers in the English language were included and therefore relevant papers may have been missed due to inability to translate. Meta‐analysis was not feasible due to the heterogeneity of the data. A lack of data on safety and clinical outcomes meant that this review could not conclude if the interventions are of benefit clinically.

## 6 Conclusion

This review highlights the limited research available on deprescribing interventions targeting anticholinergic medications within hospital settings. Findings suggest that such interventions are effective in reducing anticholinergic burden scores. However, the safety and effect of this reduction on clinical outcomes remain uncertain. To address this gap, robust RCTs with extended follow‐up periods are needed to evaluate the short and long‐term clinical impact. Future efforts should also prioritise patient involvement and education to support shared decision‐making to enhance the success of deprescribing initiatives.

## Conflicts of Interest

The authors declare no conflicts of interest.

## Data Availability

Data sharing not applicable to this article as no datasets were generated or analysed during the current study.

## Appendix 1

### Search Keywords and MeSH Terms Used

**Table:**

Population	Hospitalised	Intervention	Outcome
Elderly		Deprescribing	Anticholinergic/anticholinergic burden
(elderly filter broad) exp aged/ or exp geriatrics/ or exp geriatric nursing/or (centarian* or centenarian* or elder* or eldest or frail* or geriatri* or nonagenarian* or octagenarian* or octogenarian* or old age* or older adult* or older age* or older female* or older male* or older man or older men or older patient* or older people or older person* or older population or older subject* or older woman or older women or oldest old* or senior* or senium or septuagenarian* or supercentenarian* or very old*).ti,ab,kf.	in hospital.mp hospital*.mp hospital ward.mp inpatient*.mp in‐patient*.mp hospitali?ed.mp Hospitalization/	((dose or drug or medication) adj3 (withdraw* or taper* or reduc* or stop* or cessation or ceas*)).mp Medication review.mp Drug review.mp Medic* optimisation.mp Deprescrib*.mp De‐prescrib*.mp Deprescription.mp De‐prescription.mp Inappropriate ADJ3 prescribing.mp Drug uti?ation.mp Polypharmacy.mp deprescriptions/ or drug prescriptions/ or drug tapering/ or inappropriate prescribing/ or medication errors/or polypharmacy/	Anticholinergic*.mp Anti‐cholinergic*.mp Cholinergic antagonist.mp Antimuscarinic.mp Anti‐muscarinic.mp Parasympatholytic.mp Choline receptor blocking agent.mp Acetylcholine antagonist.mp Anticholinergic Syndrome/or exp Cholinergic Antagonists/
